# Pharmacovigilance in children in Camagüey Province, Cuba

**DOI:** 10.1007/s00228-012-1222-9

**Published:** 2012-02-08

**Authors:** Z. Bárzaga Arencibia, A. López Leyva, Y. Mejías Peña, A. R. González Reyes, E. Fernández Manzano, I. Choonara

**Affiliations:** 1 Children’s Hospital “Eduardo Agramonte Piña”, Camagüey Province, Cuba; 2 Provincial Pharmacoepidemiology Department, Camagüey Province, Cuba; 3 Faculty of Pharmacy, University of Havana, Havana, Cuba; 4 Academic Division of Child Health, University of Nottingham, Derbyshire Children’s Hospital, Derby, UK; 5 Academic Division of Child Health, The Medical School, University of Nottingham, Derbyshire Children’s Hospital, Uttoxeter Road, Derby, DE22 3DT UK

**Keywords:** Adverse drug reaction, Drug toxicity, Children, Cuba, Pharmacovigilance

## Abstract

**Purpose:**

Our aim was to describe the adverse drug reactions (ADRs) detected following increased education about pharmacovigilance and drug toxicity in children in Camagüey Province, Cuba.

**Methods:**

Over a period of 24 months (January 2009 to December 2010), all reports of suspected ADRs in children to the Provincial Pharmacovigilance Centre in Camagüey Province were analysed. ADRs were classified in relation to causality and severity.

**Results:**

There were 533 reports involving suspected ADRs in children in the period. Almost one third of the reports received were classified as moderate (155, 29%) or severe (10, 2%). There was one fatality in association with the use of ceftriaxone. Vaccines and antibiotics were responsible for most of the ADR reports (392, 74%) and for all ten severe ADRs. After an intensive educational package, both within the community and the Children’s Hospital, the number of reports increased from 124 in 2008 to 161 in 2009 and 372 in 2010. This was equivalent to a reporting rate of 879 and 2,031 reports per million children per year for 2009 and 2010, respectively.

**Conclusions:**

The incidence of ADRs in children Camagüey Province, Cuba, is greater than previously reported. An educational intervention about pharmacovigilance and drug toxicity in children can improve the reporting of ADRs.

## Introduction

Adverse drug reactions (ADRs) are a significant problem in children, and it is widely accepted that most are unreported. We previously demonstrated an ADR reporting rate of 634 per million children per year in Cuba [1]. Others have described 226 reports per million children per year in Sweden [2]. Systematic reviews have shown that almost one in ten children in hospital will experience an ADR [3, 4]. Although the majority of ADRs are mild, a small number are severe [5, 6]. Cuba has an excellent healthcare system, which includes an extensive primary health care system [7]. Following assessment in primary care, children may be seen in either polyclinics or in children’s hospitals. It also has a National Pharmacovigilance Programme, which was established in Cuba in 1996 [8]. Suspected ADRs are reported to provinces, which then forward them on to the National Coordinating Centre [8]. Camagüey Province has a particular interest in children who experience ADRs.

We described the ADRs detected in children in Camagüey Province in 2008 [1]. Although we demonstrated an ADR reporting rate of 634 per million children per year in Cuba, we recognised that this figure was likely to be a significant underestimate of the true rate of ADRs. We received very few reports from the Children’s Hospital in Camagüey. Additionally, the Provincial Pharmacovigilance Centre only received reports from the Municipality of Camagüey and not from the 12 other municipalities within the province. An educational package about pharmacovigilance and drug toxicity was targeted at health professionals in the province who were involved in the care of children either within the community or in hospital. This involved educational meetings, group discussion of clinical cases with suspected ADRs, individualised teaching and emails with safety alerts or specific articles focussing on pharmacovigilance/drug toxicity. The educational package recognised that different health professionals had different levels of knowledge about the subject area. In all the educational activities, there was a strong focus on the importance of reporting ADRs and an open opportunity whereby individual health professionals could ask questions and obtain appropriate practical answers. Within the hospital, intensive education was provided to complement the Drug and Therapeutics Committee to emphasise the importance of pharmacovigilance. Here we describe the ADRs detected following increased education about pharmacovigilance and drug toxicity.

## Methods

Camagüey Province is in the central part of Cuba and includes the city of Camagüey, smaller towns and rural areas. It consists of 13 municipal areas and two children’s hospitals. The main children’s hospital, with 400 beds, is based in Camagüey, but there is also a smaller children’s hospital in one of the small towns on the edge of the province (Florida), with 75 beds. Suspected ADRs are reported to the Provincial Pharmacovigilance Centre in Camagüey either personally, by phone or by e-mail. Camagüey Province has a population of 783,372, including 183,105 children (0–18 years). Approximately 40% of the total population lives in the municipality of Camagüey, and 36% of the children of Camagüey Province live in this municipality.

Over a period of 24 months (January 2009–December 2010), all reported suspected ADR for children were analysed. Each report contained details of the age and sex of the patient, name of the health professional notifying the ADR, healthcare setting, suspected ADR, suspected medicine responsible for the ADR, reason for prescribing the medication and time between the ADR and notification. Each report was analysed in relation to ADR severity and causality and collated. Each suspected ADR was analysed by a group of health professionals trained in pharmacovigilance (the Provincial Pharmacovigilance Group of Experts). Each report was assessed using the Karch-Lasagna algorithm [9, 10]. For children who experienced more than one ADR, the clinical impact of the separate ADRs was considered together and one severity classification was given. Severity was classified as severe/moderate/mild [1, 11].

- severe: fatal or potentially life threatening or causing permanent disability;
- moderate: requiring treatment or prolonging stay in hospital or causing interference with normal daily activities;
- mild: minor reactions that do not require treatment and do not prolong stay in hospital.

## Results

In 2009, there were 161 reports of suspected ADRs in children received by the Provincial Pharmacovigilance Centre. In 2010, this increased to 372 reports, making a total of 533 reports over the 2 years. During this 2-year period, reports were received from nine of the 13 municipalities in the province. The vast majority (438, 82%) were from Camagüey Municipality. Following assessment by the Provincial Pharmacovigilance Group, the majority of ADR reports in both years (509) were classified as probable. Thirteen were considered possible, six conditional, one definite and four thought to be unlikely based on what is known about the drug. The ages of the children for whom ADRs were reported to be caused by vaccines and medicines are shown in Table 1. Over the 2 years, received reports involved 281 (53%) girls and 252 (47%) boys. Vaccines were responsible for more than half of those ADR reports(283, 53%). There was a significant increase in the number of ADR reports to vaccines in 2010 (from 75 in 2009 to 208 in 2010) and was associated with the widespread use of A-H1N1 vaccine. A total of 250 ADR reports were received for medicines; 86 of these were received in 2009, and 164 in 2010.

**Table 1:** Ages of children for whom an adverse drug reaction (ADR) was reported: 2009–2010

Age (years)	ADRs due to medicines		ADRs due to vaccines		Total	
	*n*	%	*n*	%	*n*	%
0–1 month	6	2	3	1	9	2
2–11 months	14	6	117	41	131	25
1–2 years	33	13	57	20	90	17
3–5 years	47	19	50	18	97	18
6–10 years	41	16	26	9	67	12
11–17 years	109	44	30	11	139	26
Total	250	100	283	100	533	100

The majority of reports sent to the centre over the 2 years came from primary health care (405, 76%). 128 (24%) reports came from hospitals, 11 of which were submitted in 2009 and 117 in 2010. Doctors sent most reports (390, 73%). Other reports were received from pharmacists (82, 15%), registered nurses (48, 9%), nurse technicians (7, 1.3%) and pharmacy technicians (2, 0.4%). In 2010, for the first time, the provincial centre received four ADR reports from parents (0.7%). There were 855 suspected ADRs within the reports (1.6 suspected ADRs per report). There were 490 suspected ADRs reported following the use of vaccines. The ten most frequently reported ADRs to vaccines are shown in Table 2. There were 365 separate suspected ADRs following the use of medicines, and the ten most frequently reported ADRs are shown in Table 3. A total of 58 different medicines were suspected to be responsible for the ADRs in 2009, and ten other medicines were prescribed concurrently. In 2010, 49 different medicines were suspected to be responsible for the ADRs, and 19 other medicines were prescribed concurrently.

**Table 2:** Ten most frequently reported adverse drug reactions (ADRs) to vaccines in children in Camagüey Province, Cuba: 2009–2010

ADR	2009		2010		Combined	
	*n*	%	*n*	%	*n*	%
Fever	56	44	157	43	213	43
Rhinitis/cough	0	0	47	13	47	10
Vomiting	6	5	23	6	29	6
Urticaria/angioedema	18	14	10	3	28	6
Pain/inflammation/infection at the injection site	10	8	18	5	28	6
Irritability and tearfulness	13	10	4	1	17	4
Diarrhoea	2	2	15	4	17	4
Respiratory tract infection	0	0	15	4	15	3
Headache	0	0	14	4	14	3
Peripheral cyanosis	4	3	5	1	9	2
Others	18	14	55	15	73	15
Total	127	100	363	100	490	100

**Table 3:** Ten most frequently reported adverse drug reactions (ADRs) to drugs reported in children in Camagüey Province, Cuba: 2009–2010

ADR	2009		2010		Combined	
	*n*	%	*n*	%	*n*	%
Urticaria/angioedema	41	30	64	28	105	29
Drowsiness	3	2	38	17	41	11
Vomiting	11	8	21	9	32	9
Nausea	9	7	8	4	17	5
Oedema	5	4	11	5	16	4
Irritability and tearfulness	4	3	10	4	14	4
Abdominal pain	8	6	6	3	14	4
Dizziness/vertigo	6	4	6	3	12	3
Tachycardia	4	3	6	3	10	3
Asthenia	5	4	5	2	10	3
Others	41	30	53	23	94	26
Total	137	100	228	100	365	100

The three other groups of medicines associated with a large number of ADR reports were antibiotics, antihistamines and nonopioid analgesics (Table 4). One hundred and nine reports were received for antibiotics, for a total of 159 suspected ADRs. The antibiotics most frequently associated with ADRs were benzylpenicillin (31), amoxicillin (26), erythromycin (20), azithromycin (19) and cephalexin (19). Two antihistamines, loratadine (28) and ketotifen (20), were associated with >76% of the 63 ADRs contained within 54 reports due to antihistamines. There were 35 reports of ADRs due to nonopioid analgesics, and within these reports were 50 suspected ADRs. Thirty-two of these were associated with the use of dipyrone. The most frequent ADRs following dipyrone were urticaria/angioedema (10), facial oedema (6), nausea (3) and syncope (3). Twelve ADRs were associated with the use of nonsteroidal anti-inflammatory drugs (5 ibuprofen, 3 indomethacin, 2 salicylates and 2 piroxicam) and six due to paracetamol.

**Table 4:** Drug classes and reports of suspected adverse drug reactions (ADRs)

Medicine	2009		2010		Combined	
	*n*	(%)	*n*	(%)	*n*	(%)
Antibiotics	36	42	73	45	109	44
Antihistamines	9	10	45	27	54	22
Analgesics nonopioid	11	13	24	15	35	14
Bronchodilators	6	7	8	5	14	6
Vitamins and minerals	7	8	2	1	9	4
Prokinetic agents	2	2	4	2	6	2
Antiparasitic agents	5	6	0	0	5	2
Corticosteroids	2	2	1	1	3	1
Others	8	9	7	4	15	6
Total	86	100	164	100	250	100

The majority of reports received were classified as mild (367, 69%); 155 (29%) were moderate and ten (2%) were severe. There was one fatality in association with the use of ceftriaxone. Vaccines (7) and antibiotics (3) were responsible for the ten severe ADRs (Tables 5 and 6).

**Table 5:** Severe adverse drug reactions (ADRs) to vaccines: 2009–2010

Year	Age	Sex	Drug	ADR	Comments
2009	5 months	M	Pentavalent vaccine	Diarrhoea, vomiting, dehydration resulting in shock	
	5 months	F	Pentavalent vaccine	Vomiting, fever resulting in shock	
	8 months	F	Pentavalent vaccine	Deep venous thrombosis	Bilateral renal hypoplasia
	5 months	F	Pentavalent vaccine	Seizure	
	7 months	M	Pentavalent vaccine	Seizure	
2010	3 years	F	A-H1N1 vaccine	Severe bronchopneumonia resulting in shock	Epilepsy
	3 years	M	A-H1N1 vaccine	Fever, asthenia, vomiting resulting in shock

**Table 6:** Severe adverse drug reactions (ADRs) to medicines: 2009–2010

Year	Age	Sex	Drug	ADR	Comments	Onset of ADR in relation to drug	Indications
2009	1 month	F	Ceftriaxone	Anaphylactic shock	Patient died during the first IV dose	During administration	Acute infectious diarrhoea
	4 years	F	Amoxicillin	Seizure		Within 24 h	Tonsillitis
2010	3 years	M	Sodium benzyl penicillin & procaine benzyl penicillin	Seizure	Fifth IV dose. Other medicines: prednisolone, theophylline, vitamin C, cough medicine	Within 1 h	Bronchopneumonia

The clinical impact of the ADR was significant in 60 children in 2009 in that they required emergency care either in a polyclinic or a hospital. In 2010, a similar number (54) required emergency care. In 2008, 19 moderate and seven severe ADRs and 20 children required emergency treatment in a polyclinic or a hospital [1].

## Discussion

Underreporting of ADRs is a significant problem worldwide. There is significant variation between different countries in relation to the number of ADRs reported. The Uppsala Monitoring Centre receives data from national pharmacovigilance centres of >100 countries. During 2010, the centre received >1 million reports, with New Zealand submitting the highest number of reports per capita (1,200 reports per 1 million inhabitants per year) [12]. Cuba submits the highest number of reports per capita in Latin America [12]. 

We previously demonstrated an ADR reporting rate of 634 per 1 million children per year in Cuba [1], which is considerably higher than the reporting rate from Sweden of 226 per 1 million children per year [2] and Denmark of 222 per 1 million children per year [13]. Following an intensive educational package, both within the community and the Children’s Hospital, we managed to significantly increase the reporting rate of ADRs. The number of reports in Camagüey Province per 1 million children per year in 2009 was 879 and in 2010 was 2,031. The number of reports was considerably higher than the reports received in Cuba nationally in 2009 and 2010: 677 and 1,278 reports per 1 million children per year, respectively. It is important to recognise that the increased reporting of ADRs detected a greater proportion of moderate and severe ADRs (31%) than prior to the educational intervention (21%) [1]. The most frequently reported ADRs in our study (fever, urticaria and vomiting) were similar to the most frequently reported ADRs in a recent paper from the Uppsala Monitoring Centre, which describes suspected ADRs reported for children worldwide [14]. The Children’s Hospital in Camagüey is the main children’s hospital in the province and is a referral centre for paediatric intensive care, oncology, neurosurgery and nephrology alongside general paediatrics. It has had a Drug and Therapeutics Committee (DTC) since 1998 and since then has been active in promoting more rational use of medicines [15]. In 2008 and 2009, there were only 12 and 11 reports received from the hospital, respectively. In 2010, following the interventions, there was a tenfold increase to 117. This had only been possible due to the combined efforts of all members of the Drug and Therapeutics Committee emphasising the importance of pharmacovigilance. It is recognised, however, that this reporting rate is still likely to be a significant underestimate of the number of ADRs. Cytotoxic agents and anticonvulsants are recognised as the group of medicines most likely to be associated with ADRs in children [5, 16]. Despite the educational intervention, however, there were no reports of ADRs to cytotoxic agents and only two for anticonvulsants in 2009 and 2010. ADRs to cytotoxic agents and anticonvulsants are often severe [5], and it is important, therefore, that these ADRs are both suspected and reported. Specific discussions with the health professionals involved in treating children with malignancies and epilepsy may be beneficial in encouraging the submission of ADR reports from these two groups of children.

Reports were received from the majority of the 13 municipalities in the province. There are practical difficulties in communicating the reports of suspected ADRs in a lower–middle income country. The postal system is unreliable, and there are often shortages of forms for suspected ADRs. Postal reports are therefore less likely from municipalities outside of the city of Camagüey. Additionally, in these predominantly rural areas, one is more likely to experience problems with Internet connections and subsequently e-mail reporting, as well as problems of inadequate signal strength for mobile phones. These problems are likely to be experienced by other national pharmacovigilance centres operating in low and lower–middle income countries.

Our study shows the benefit of an educational intervention in relation to pharmacovigilance in children. It is recognised that the number of reports received (2,031 per 1 million children per year) is, however, still an underestimate. In addition, there is a need to improve the prescribing of medicines. Ceftriaxone is not recommended in the neonatal period for two reasons: Firstly, it is highly protein bound, and this may result in the displacement of bilirubin [17]. Secondly, it has been associated with sudden death in neonates and young infants due to calcium precipitation following coadministration of calcium-containing intravenous solutions [18]. The inappropriate prescription of ceftriaxone to a neonate emphasises the need for greater awareness regarding medicine toxicity in specific age groups for all professionals involved with children. The large number of ADRs following the use of dipyrone also emphasises the need for more rational prescribing. Because of safety concerns (especially agranulocytosis and other blood dyscrasias) regarding dipyrone, the drug is not available in many countries worldwide [19, 20]. Greater awareness of the toxicity associated with the use of nonsteroidals, in particular, dipyrone, in children needs to be emphasised. A long-term aim of a pharmacovigilance programme is to ensure medicines are prescribed more rationally and thus reduce ADRs.
